# Misdiagnosed bilateral C5-C6 dislocation causing cervical spine instability: a case report

**DOI:** 10.4076/1757-1626-2-6149

**Published:** 2009-07-14

**Authors:** Ioannis D Gelalis, Georgios Christoforou, Christina M Arnaoutoglou, Angelos N Politis, Gregory Manoudis, Theodoros A Xenakis

**Affiliations:** 1 Department of Orthopaedic Surgery, University Hospital of Ioannina, St. Niarchos Avenue, 45500, Ioannina, Greece

## Abstract

**Introduction:**

The diagnosis of cervical spine injuries remains a significant problem in many blunt trauma patients. Correct and early diagnosis of these injuries is imperative as delayed or missed diagnoses result in increased morbidity and mortality.

**Case presentation:**

A 57-year-old Caucasian woman presented with a misdiagnosed bilateral C5-C6 dislocation one month after a fall and head injury, without clearance of the cervical spine in her previous visits to two physicians and having already started physiotherapy sessions, despite the presence of pain in the clinical examination. Dislocation was treated with open reduction and spinal fusion with posterior instrumentation 4 weeks post-trauma.

**Conclusions:**

Every physician should be highly suspicious of cervical spine injury in blunt trauma patients with positive clinical examination and include radiologic studies in his screening modality. Physiotherapy sessions should under no circumstances be started in the presence of underlying spine injury.

## Case presentation

A 57-year-old Caucasian female presented to our emergency department complaining of neck pain one month after having a fall from a height and an injury on her occipital bone. The patient was non-smoking, non-drinking and with a free medical history. She weighed 61 kg and had a height of 162 cm.

The patient had already visited two physicians. The first assessment was made at the day of the injury at a hospital elsewhere. This first clinical examination revealed pain at the cervical spine and deficiency in the range of the cervical spine motion but no neurologic impairment was present. No radiologic examination was ordered and the patient received analgesics and recommendation for physiotherapy sessions. Persistence of pain led to a second assessment by another physician one week later at another hospital. Analgesics were once more prescribed and radiological screening was once more not considered essential.

**Figure 1:**
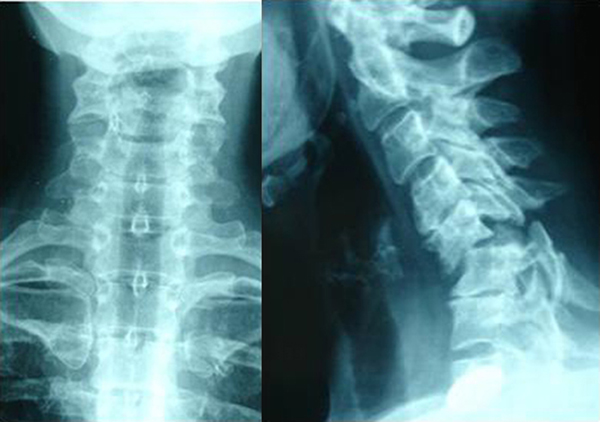
**Anteroposterior and lateral X-ray showing bilateral C5-C6 dislocation**.

**Figure 2:**
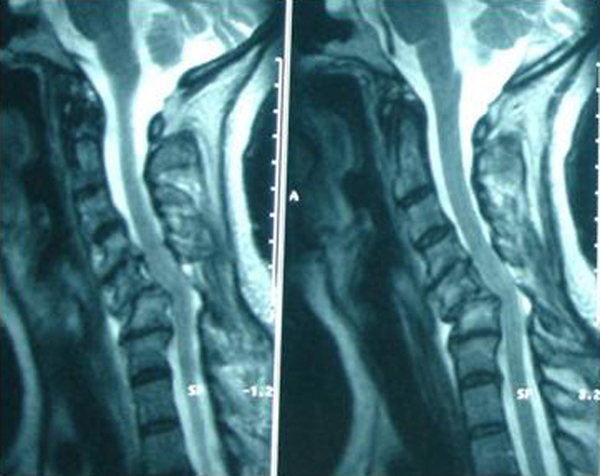
**MRI screening showing anterior angulation and narrowing of the spinal canal**.

On examination in our department, the patient showed no neurologic deficit, whereas restriction of range of motion and pain remained. Three-view radiographs (lateral, anteroposterior and odondoid) ordered by the attending emergency physician showed bilateral C5-C6 dislocation causing instability (Figure 1). CT and MRI scan showed olisthesis of C5 over C6, anterior angulation and narrowing of the spinal canal without spinal cord pressure from disc material (Figure 2). The electrophysiological testing was normal.

**Figure 3:**
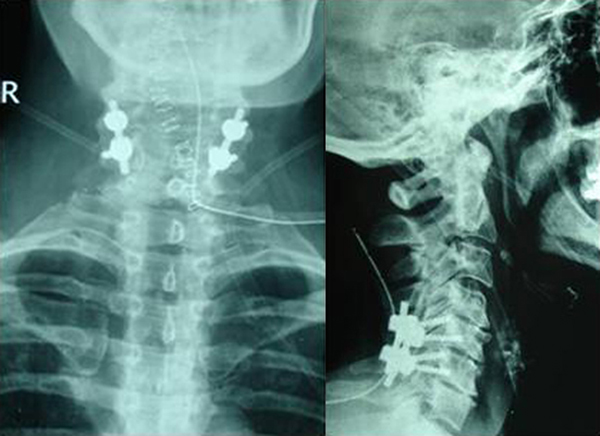
**Postoperative X-rays**.

The patient was transferred to the operating theatre at the day of the admission. Open reduction of the bilateral dislocation and spinal fusion with posterior instrumentation were performed under continuous intraoperative neurophysiological monitoring (Figure 3). Duration of hospitalization was four days. Mobilization began the second postoperative day and the patient was discharged using a Philadelphia collar. After twelve months of follow up, the patient reported free of complaints and with excellent return to her every day activities.

## Discussion

Prompt and accurate diagnosis of cervical spine injuries is crucial in preventing the devastating consequences of undetected fractures or dislocations. Up to 30% of these patients may suffer permanent neurologic sequelae [1].

For blunt trauma patients with Glasgow Coma Scale greater than 13, a negative clinical examination of the cervical spine is a better screening modality than lateral radiographs. If symptoms are elicited, radiologic studies are warranted [2]. All trauma patients with a complaint of mild neck pain require radiological examination of the cervical spine, even if their neurological examination is normal. A standard three-view series must be obtained and it is important to ensure that the plain radiographs demonstrate all seven cervical vertebrae and the top of the first thoracic vertebra before reading them [3]. When adequate flexion and extension motion is present, the flexion and extension examination for the acute evaluation of blunt trauma has a very low false-negative rate for ligamentous injuries. In the acute setting, however, 30% of blunt trauma patients have a limited ability to flex and extend the cervical spine. Flexion and extension radiographs may yield false-negative results in such cases. These patients are at increased risk for injury and cross-sectional imaging is suggested [4].

With the recent development of newer generation high speed CT scanners, cervical spine CT scanning is being utilized with increasing frequency as a screening test. The sensitivity for plain radiography for detecting patients with cervical spine injury is 52%, while the sensitivity for computed tomography scanning is 98%. Thus, CT significantly outperforms plain radiography as a screening test for patients at very high risk of cervical spine injury and should be the initial screening test in patients with a significantly depressed mental status [5]. CT may fail to identify horizontal fractures or those obscured by artifacts from dental work, particularly at C2 and should therefore be combined with a single lateral radiograph [6]. Ligamentous injuries may only be detected with magnetic resonance imaging (MRI).

## Conclusions

Diagnostic imaging of the cervical spine is essential in the evaluation of every symptomatic trauma patient. In our case the patient continued her everyday activities sustaining a bilateral cervical spine dislocation for one month. Moreover, physiotherapy sessions were recommended, increasing the risk for irreversible neurologic sequelae. Emergency department doctors should be strict in following the pre-mentioned evaluation protocols. Immediate immobilization of the cervical spine and operative treatment was the appropriate treatment algorithm in our case.

## Abbreviations

CT: Computed tomography; MRI: Magnetic resonance imaging.

## Consent

Written informed consent was obtained from the patient for publication of this case report and accompanying images. A copy of the written consent is available for review by the Editor-in-Chief of this journal.

## Competing interests

The authors declare that they have no competing interests.

## Authors' contributions

GI and XT developed the concept and wrote the draft. CG, PA and MG reviewed the images and contributed in writing the manuscript. XT, GI and CA performed the surgical procedure. All authors contributed to patient care. All authors read and approved the final manuscript.
